# Pretreatment absolute lymphocyte count is an independent predictor for survival outcomes for esophageal squamous cell carcinoma patients treated with neoadjuvant chemoradiotherapy and pembrolizumab: An analysis from a prospective cohort

**DOI:** 10.1111/1759-7714.14898

**Published:** 2023-04-24

**Authors:** Wei‐Xiang Qi, Xiaoyan Wang, Chengqiang Li, Shuyan Li, Huan Li, Feifei Xu, Jiayi Chen, Shengguang Zhao, Hecheng Li

**Affiliations:** ^1^ Department of Radiation Oncology, Ruijin Hospital Shanghai Jiaotong University School of Medicine Shanghai China; ^2^ Department of Radiation Oncology The Third Affiliated Hospital of Wenzhou Medical University Wenzhou China; ^3^ Department of Thoracic Surgery, Ruijin Hospital Shanghai Jiaotong University School of Medicine Shanghai China

**Keywords:** absolute lymphocyte count, esophageal squamous cell carcinoma, neoadjuvant chemoradiotherapy, pathological complete response, pembrolizumab

## Abstract

**Background:**

The aim of the study was to analyze the relationship between pretreatment inflammatory biomarkers (IBs) and survival outcomes for patients with esophageal squamous cell carcinoma (ESCC) treated with neoadjuvant chemoradiotherapy (neo‐CRT) and pembrolizumab.

**Methods:**

Clinical variables and IBs (absolute monocyte count [AMC], absolute lymphocyte count [ALC], platelet count [PLT], neutrophil‐to‐lymphocyte ratio [NLR], platelet‐to‐lymphocyte ratio [PLR], lymphocyte‐to‐monocyte ratio [LMR], pan‐immune inflammation value [PIV], systemic immunoinflammatory index [SII], systemic immunoreactivity index [SIRI] and prognostic nutritional index [PNI]) were collected. Univariate and multivariate analysis were performed to identify the independent factors for outcomes of ESCC.

**Results:**

A total of 51 patients were included. Of these, 35 patients achieved pathological complete response (pCR) after neo‐CRT and pembrolizumab (pCR: 68.6%). With a median follow‐up of 20 months, the two‐year PFS and OS of the cohort was 64% and 91%, respectively. Multivariate logistic regression analysis indicated that ALC (overall response [OR] 4.4, *p* = 0.051) and PLT (OR 6.7, *p* = 0.023) were two independent predictors for achieving pCR among ESCC treated with neo‐CRT and pembrolizumab. Multivariate Cox regression analysis showed that ALC (HR 0.27, *p* = 0.028) and SIRI (HR 3.13, *p* = 0.048) were two independent predictors associated with PFS. Kaplan Meier analysis demonstrated that the PFS of ESCC with high baseline ALC was significantly better than those with low ALC (2‐year PFS: 77% vs. 47%, *p* = 0.027), but not for overall survival (2‐year OS: 96% vs. 87%, *p* = 0.46).

**Conclusions:**

This retrospective analysis based on a prospective cohort for the first time demonstrates that pretreatment ALC is an independent predictor for achieving pCR and favorable outcomes of ESCC treated with neo‐CRT and pembrolizumab.

## Graphical abstract

Baseline absolute lymphocyte count (ALC) is associated with PFS among ESCC treated with neo‐CRT and pembrolizumab.

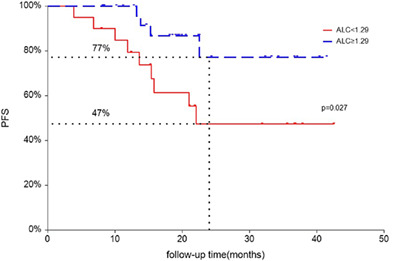

## INTRODUCTION

According to the 2020 global cancer statistics, esophageal cancer ranks as the seventh most commonly diagnosed cancer, accounting for 604 000 new cases and the sixth in mortality with 544 000 deaths around the world.^1^ In China, about 90% of the histological types are esophageal squamous cell carcinoma (ESCC). Since the publication of the CROSS^2^, ^3^ and NEOCRTEC 5010 trials,^4^, ^5^ neoadjuvant chemoradiotherapy (neo‐CRT) followed by curative surgery has become the standardized treatment for locally advanced esophageal cancer (LAEC). However, the prognosis for patients with LAEC remains poor with a 10‐year overall survival of 38%, and more than half of these patients develop cancer recurrence.^6^ Therefore, there is an urgent need to develop novel and efficient treatments to improve the long‐term outcomes of LAEC patients.

In recent years, immunotherapies have revolutionized the treatment of advanced solid tumors including esophageal cancer. Among them, the PD‐1/PD‐L1 pathway is a major checkpoint pathway for immune responses, which has been a common mechanism of immune escape used by tumor cells.^7^, ^8^ Therefore, multiple phase III trials have been conducted to investigate the efficacy and safety of immune checkpoint inhibitors (ICIs) for the treatment of LAEC. Indeed, several large phase III trials, including ATTRACTION‐3, CheckMate 648, KEYNOTE‐181 and KEYNOTE‐590, have confirmed that ICIs alone, or combined with chemotherapy as first‐ or second‐line therapy, significantly improved the outcomes of LAEC when compared to controls.^9^, ^10^, ^11^, ^12^ However, the role of ICIs as neoadjuvant treatment for ESCC remains unknown. We thus performed a prospective phase I trial (PALACE‐1) to investigate the efficacy and toxicity of neoadjuvant CRT combined pembrolizumab for LAEC, and the addition of ICIs to neo‐CRT induced a pathological complete response (pCR) of 55.6% for resected tumors.^9^ A phase II multicenter study (PALACE‐2) is also underway to further confirm our findings.

Prior to the present study, multiple studies have been performed and demonstrated that the inflammation‐based index, such as the neutrophil‐to‐lymphocyte ratio (NLR),^10^, ^11^, ^12^, ^13^, ^14^ platelet‐to‐lymphocyte ratio (PLR),^15^, ^16^, ^17^ and prognostic nutritional index (PNI)^18^, ^19^, ^20^ are significantly associated with outcomes in LAEC patients treated with neoadjuvant CRT. More recently, Wu et al.^21^ performed a retrospective study and found that NLR at 6 weeks post treatment is a predictor of the response to anti‐PD‐1 treatment in patients with ESCC. However, there is a lack of studies which have assessed the relationship between hematological biomarkers and outcomes of ESCC patients treated with neo‐CRT with ICIs. As a result, we performed the present study to comprehensively identify hematological biomarkers associated with outcomes of ESCC treatment with neo‐CRT with ICIs in order to tailor the treatments.

## METHODS

### Data collection

A total of 51 patients with ESCC treated with CRT and pembrolizumab were prospectively collected at the Department of Radiation Oncology, Ruijin Hospital, Shanghai Jiaotong University School of Medicine between March 2019 and March 2022 in two prospective trials (PALACE‐1, NCT04435197l; part of enrolled patients in PALACE‐2: NCT04435197), and approved by the ethics committee of Ruijin Hospital. The latest follow‐up time was June 20, 2022. All procedures were conducted according to the institutional and national standards on human experimentation and with the declaration of Helsinki of 1964.

The inclusion and exclusion criteria have been reported in our previous study.^9^ Medical records were reviewed and the following information including age at diagnosis, baseline ECOG performance status, sex, baseline weight and height and smoking/drinking history, comorbidities, BMI (body mass index), tumor location of ESCC (upper, middle, lower segment), tumor length (cm), histological types, primary tumor (T) stage, regional lymph nodes (N) stage, distant metastasis (M) stage, clinical TNM stage according to eighth AJCC, start and completion of neo‐CRT, surgery time, time interval between completion of neo‐CRT end and surgery, the peripheral inflammatory biomarkers (IBs) before treatment including baseline absolute neutrophil count, absolute lymphocyte count (ALC), absolute monocyte count (AMC), absolute platelet count and absolute leukocyte count, albumin (g/L), and C reactive protein (CRP) were recorded. Moreover, the pathological results and the pCR status after surgery was recorded. The primary outcome of the present study was to identify the independent IBs for predicting pCR, which was defined as no evidence of vital residual tumor cells. The secondary outcome of the study was to identify the independent IBs for predicting favorable progression‐free survival (PFS), which was defined as the time from diagnosis of esophageal cancer to the time of disease progression or death/last follow‐up.

### Neoadjuvant CRT regimen

The chemotherapy regimen consisted of carboplatin (area under the curve of 2 mg/mL per min) and paclitaxel (50 mg/m^2^ of body surface area), which were administered intravenously on days 1, 8, 15, 22. Concurrent radiotherapy was performed on day 1 of chemotherapy with a total dose of 41.4 Gy in 23 fractions, on five fractions per week. The gross tumor volume (GTV) was determined by the contrast‐enhanced CT, barium swallow, endoscopic examination, or PET‐CT, which contained both the primary tumor (GTVp) and the positive regional lymph nodes (GTVn). The clinical target volume included the GTVp with an additional 3.0‐cm proximal and distal margin and 0.5 cm radial margin, as well as GTVn with 0.5 cm uniform margin. The planning target volume was expanded to include a 0.5 cm margin from the clinical target volume for setup variations and respiratory‐induced tumor motion. Pembrolizumab was given on days 1 and 22 of the neoadjuvant therapy at a dose of 200 mg. After the completion of neoadjuvant treatment, all patients received curative surgery excluding those patients where there was a contraindication to surgery. During the first year after treatment completion, patients were followed‐up every 3 months, and once every 6 months thereafter.

### Definition of inflammatory biomarkers

All hematological examinations were tested before neo‐CRT and pembrolizumab. Inflammatory biomarkers (IBs) were defined as follows: NLR = (the ratio of neutrophil count to lymphocyte count);^22^ LMR = (the ratio of lymphocyte count to monocyte count);^23^ PLR = (the ratio of platelet count to lymphocyte count).^24^ The PNI was the sum of albumin value (g/L) and five times lymphocyte count (10^9^/L);^25^ SII = (platelet count) × NLR;^26^, ^27^ SIRI = neutrophils × monocytes/lymphocytes;^28^ PIV = (neutrophil count × platelet count × monocyte count)/lymphocyte count^29^, ^30^ BMI = weight (kg)/height (m)^2^.^31^

### Statistical analysis

The baseline characteristics of included patients were simply described by using frequencies and percentages. Continuous variables of IBs were converted into binary variables by measuring the area under the receiver operating characteristic (ROC) curve. Univariate and multivariate logistic‐regression analyses were performed to investigate predictors for pCR. Univariate and multivariate Cox proportional hazards regression was performed for identifying factors associated with PFS. Kaplan–Meier method was used to estimate PFS, and the log‐rank test was applied to test statistical significance. All statistical analyses were conducted through MedCalc version 19.6.4 statistical software.

## RESULTS

### Patient characteristics

The baseline characteristics of patients are shown in Table 1. All patients received neo‐CRT combined with pembrolizumab before radical surgery. Of these, the median age of patients was 62 years, and 51 (100%) had a performance status of 1 or less. There were 44 male and seven female patients. As for smoking histology, 13 patients had no history of smoking, while 38 patients had a history of smoking. All 51 ESCC patients were treated with esophagectomy after neoadjuvant treatment. Of these, 35 patients achieved pCR (68.6%). A total of 44 patients (86.3%) achieved disease progression‐free survival until the last follow‐up on June 20, 2022 (Table 1); the two‐year PFS and OS of the cohort was 64% and 91%, respectively.

**TABLE 1:** Baseline characteristics of included patients.

Variable	No. of patients	%
Age		
Median, (range)	62 (39–75) years	
Sex		
Male	44	86.3
Female	7	13.7
Histology		
SCC	51	100
Comorbidities		
Yes	22	43.1
No	29	56.9
Smoking		
Yes	38	74.5
No	13	25.5
Drinking		
Yes	32	62.7
No	19	37.3
Tumor location		
Upper thoracic	11	21.6
Middle thoracic	13	25.5
Low thoracic	27	52.9
Tumor length, cm	4.7 (range: 1.2–14 cm)	
cT stage		
T3	43	84.3
T4	8	15.7
cN stage		
N0–1	18	35.3
N2–3	33	64.7
Stage		
II–IIIA	16	31.4
IIIB	20	39.2
IVA	15	29.4
ECOG status		
0	26	51
1	25	49
BMI	22.83 (range: 17.1–28.93)	‐
Time interval from radiation to surgery, days	52 (range: 34–99)	‐
pCR		
Yes	35	68.6
No	16	31.4

Abbreviations: BMI, body mass index; LMR, lymphocyte‐to‐monocyte ratio; NLR, neutrophil‐to‐lymphocyte ratio; pCR, pathological complete response; PFS, progression‐free survival; PIV, pan‐immune inflammation value; PLR, platelet‐to‐lymphocyte ratio; PNI, prognostic nutrition index; SII, systemic immune‐inflammation index; SIRI, systemic inflammation response index.

### Optimal cutoff analysis

**FIGURE 1:**
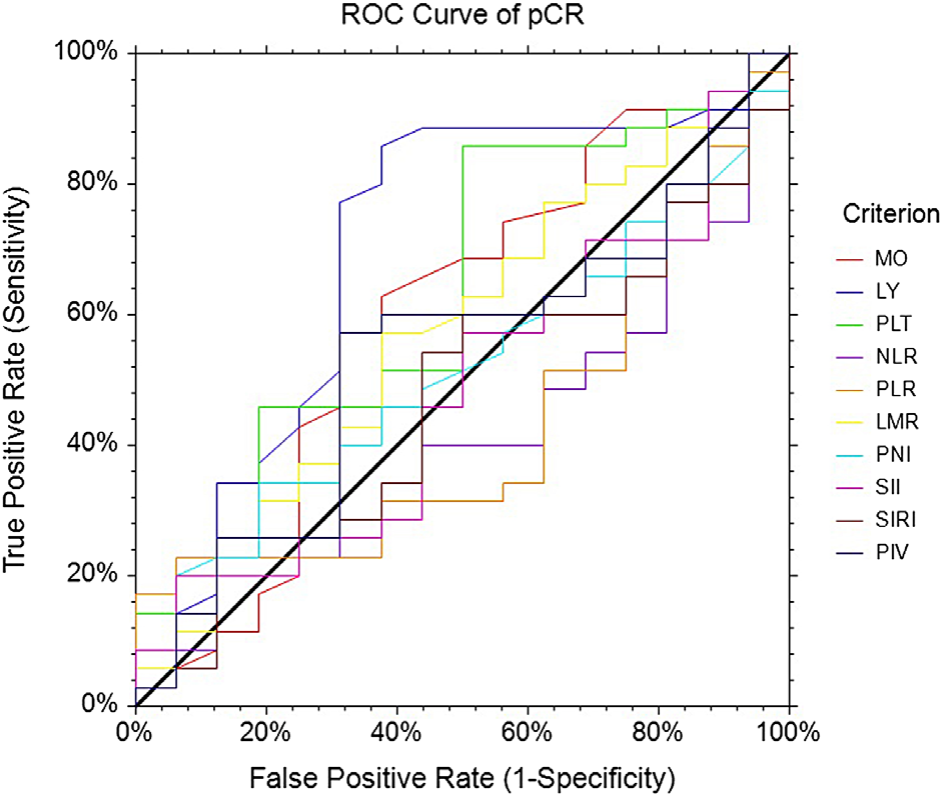
ROC curve of the preoperative inflammation markers for pCR. pCR, pathological complete response, ROC, receiver operating characteristic.

We performed ROC curve analysis to evaluate the predictive capability of these IBs for pCR and PFS. The optimal cutoff values of the ALC, AMC, PLT, NLR, PLR, LMR, PNI, SII, SIRI and PIV are listed in Table 2 and Figure 1.

**TABLE 2:** The optimal cutoff value for serum markers by ROC curves.

Serum markers	Cutoff value
Tumor length	2.8 cm
Time interval	53 days
BMI	25
LY	1.29 × 10^9^/L
Mo	0.4 × 10^9^/L
PLT	162 × 10^9^/L
NLR	2.60
PLR	150.63
LMR	3.36
PNI	52.35
SII	373.81
SIRI	0.94
PIV	232.83

Abbreviations: BMI, body mass index; LMR, lymphocyte‐to‐monocyte ratio; LY, lymphocytes; Mo, monocytes; NLR, neutrophil‐to‐lymphocyte ratio; PIV, pan‐immune inflammation value; PLR, platelet‐to‐lymphocyte ratio; PLT, platelets; PNI, prognostic nutrition index; ROC, receiver operating characteristic; SII, systemic immune‐inflammation index; SIRI, systemic inflammation response index.

### Logistic regression analysis for factors associated with pCR

Data regarding age, ECOG performance status, smoking status, white blood cell count (WBC), neuron specific enolase (NSE), lymphocytes (LY), monocytes (MO), platelets (PLT), NLR, PLR, LMR, SII, SIRI, PNI and PIV score are included in the univariate logistic regression analysis (Table 3). Univariate logistic analysis indicated that T stage (overall response [OR] 0.21, *p* = 0.051), ALC (OR 6.36, *p* = 0.0053), PLT (OR 6.0000, *p* = 0.001), and AMC (OR 2.82, *p* = 0.096) were potentially related to achieving pCR after neo‐CRT. Given the limitations of univariate analysis, multivariate logistic regression analysis was performed and indicated that ALC (OR 4.4, *p* = 0.051), and PLT (OR 6.7, *p* = 0.023) were two independent predictors for achieving pCR among ESCC patients treated with neo‐CRT and pembrolizumab (Table 3).

**TABLE 3:** Univariate and multivariate logistic analysis for factors for predicting pCR.

Factors	Univariate analysis		Multivariate analysis	
	RR, 95% CI	*p*‐value	RR, 95% CI	*p*‐value
Age				
≤60	1			
>60	2.00 (0.54–7.47)	0.30		
Sex				
Female	1			
Male	0.86 (0.15–4.97)	0.86		
BMI				
≤25	1			
>25	1.04 (0.27–4.05)	0.96		
T stage				
cT3	1		1	
cT4	0.21 (0.042–1.01)	0.051	0.13 (0.017–1.09)	0.061
N stage				
cN0–1	1			
cN2–3	0.77 (0.22–2.71)	0.68		
Stage				
III	1			
IVA	0.51 (0.15–1.76)	0.29		
Time interval				
≤53 days	1			
>53 days	3.39 (0.66–17.56)	0.15		
Tumor length				
≤2.8 cm	1			
>2.8 cm	6.00 (0.70–51.66)	0.10		
CRP				
<10	1			
≥10	2.80 (0.54–14.63)	0.22		
Absolute lymphocyte count				
<1.29 × 10^9^/L	1		1	
≥1.29 × 10^9^/L	6.36 (1.73–23.34)	0.0053	4.40(1.00–19.46)	0.051
Absolute monocyte count				
<0.4 × 10^9^/L	1		1	
≥0.4 × 10^9^/L	2.82 (0.83–9.58)	0.096	2.54 (0.53–12.05)	0.24
PLT				
<162 × 10^9^/L	1		1	
≥162 × 10^9^/L	6.00 (1.54–23.44)	0.001	6.7 (1.30–34.42)	0.023
NLR				
<2.6	1			
≥2.6	3.25 (0.78–13.48)	0.10		
PLR				
<150.6	1			
≥150.6	2.82 (0.83–9.58)	0.096		
LMR				
<3.36	1			
≥3.36	1.71 (0.52–5.65)	0.38		
PNI				
<52.35	1			
≥52.35	2.261 (0.54–9.51)	0.27		
SII				
<373.8	1			
≥373.8	2.42 (0.46–12.80)	0.30		
SIRI				
<0.94	1			
≥0.94	1.99 (0.47–8.42)	0.35		
PIV				
<232.8	1			
≥232.8	0.45 (0.13–1.51)	0.20		

Abbreviations: BMI, body mass index; CI, confidence interval; LMR, lymphocyte‐to‐monocyte ratio; NLR, neutrophil‐to‐lymphocyte ratio; pCR, pathological complete response; PFS, progression‐free survival; PIV, pan‐immune inflammation value; PLR, platelet‐to‐lymphocyte ratio; PNI, prognostic nutrition index; SII, systemic immune‐inflammation index; SIRI, systemic inflammation response index.

### Cox regression analysis for factors associated with PFS

**FIGURE 2:**
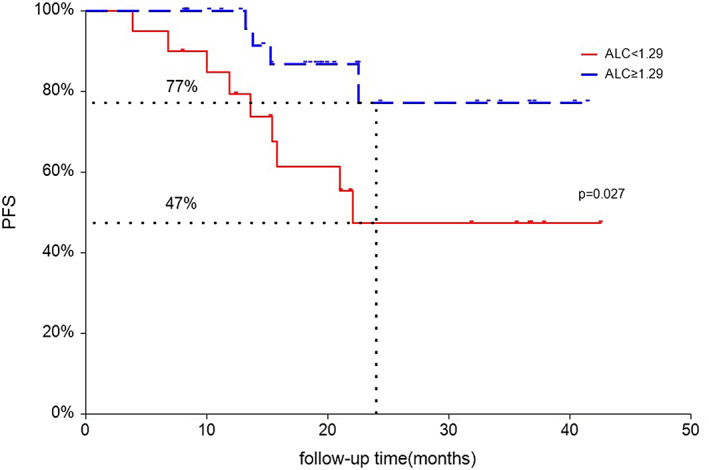
Kaplan–Meier curves for progression‐free survival (PFS) according to baseline absolute lymphocyte count (ALC). Red line indicates patients with low lymphocytes (LY) (<1.29 × 10^9^/L), and blue line indicates patients with high LY (≥1.29 × 10^9^/L).

**FIGURE 3:**
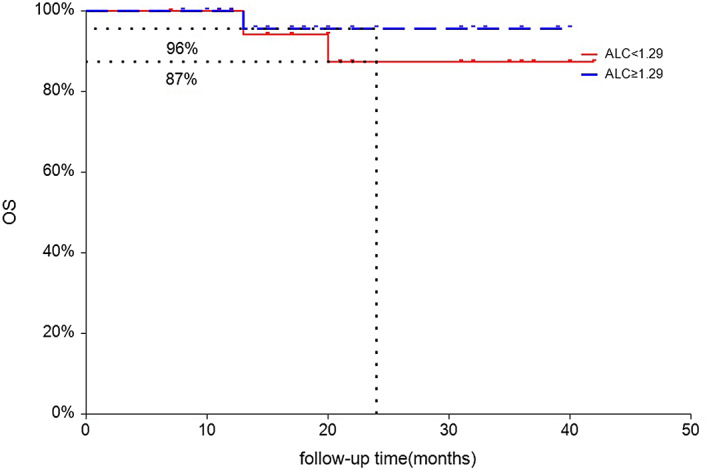
Kaplan–Meier curves for OS according to baseline absolute lymphocyte count (ALC). Red line indicates patients with low lymphocytes (LY) (<1.29 × 10^9^/L), and blue line indicates patients with high LY (≥1.29 × 10^9^/L).

Univariate Cox regression analysis showed that ALC (HR 0.29, *p* = 0.038) and SIRI (HR 2.82, *p* = 0.075) were significantly related to PFS of ESCC. Given the limitations of univariate analysis, multivariate Cox regression analysis was performed to investigate the independent factors associated with PFS (Table 4). Our findings showed that ALC (HR 0.27, *p* = 0.028) and SIRI (HR 3.13, *p* = 0.048) were two independent predictors for PFS of ESCC treated with neo‐CRT and pembrolizumab (Table 4). Kaplan Meier analysis showed that the PFS of ESCC with high level of baseline ALC (LY ≥ 1.29 × 10^9^/L) was significantly better than those with low baseline (LY <1.29 × 10^9^/L) ALC groups (2‐year PFS: 77% vs. 47%, *p* = 0.027, Figure 2), but not for overall survival (2‐year OS: 96% vs. 87%, *p* = 0.46, Figure 3).

**TABLE 4:** Univariate and multivariate Cox‐regression analysis for factors associated with PFS.

Factors	Univariate analysis		Multivariate analysis	
	HR, 95% CI	*p*‐value	HR, 95% CI	*p*‐value
Age				
≤60	1			
>60	0.81 (0.25–2.65)	0.73		
Sex				
Female	1			
Male	0.59 (0.13–2.67)	0.49		
KPS score				
90	1			
100	0.95 (0.31–2.94)	0.93		
Smoking status				
Yes	1			
No	0.48 (0.16–1.48)	0.20		
Alcohol drinking status				
No	1			
Yes	1.04 (0.34–3.19)	0.95		
BMI				
≤25	1			
>25	1.17 (0.62–6.67)	0.24		
T stage				
cT3	1			
cT4	1.15 (0.32–4.21)	0.83		
N stage				
cN0‐1	1			
cN2‐3	0.64 (0.22–1.92)	0.43		
Stage				
III	1			
IVA	1.28 (0.42–3.93)	0.66		
Time interval				
≤53 days	1			
>53 days	1.19 (0.26–5.55)	0.82		
Tumor length				
≤2.8 cm	1			
>2.8 cm	0.92 (0.20–4.17)	0.91		
CRP				
<10	1			
≥10	1.44 (0.32–6.57)	0.64		
Absolute lymphocyte count				
<1.29 × 10^9^/L	1		1	
≥1.29 × 10^9^/L	0.29 (0.088–0.94)	0.038	0.27(0.08–0.87)	0.028
Absolute monocyte count				
<0.4 × 10^9^/L	1			
≥0.4 × 10^9^/L	0.50 (0.17–1.48)	0.21		
PLT				
<162 × 10^9^/L	1			
≥162 × 10^9^/L	1.33 (0.37–4.83)	0.67		
NLR				
<2.6	1			
≥2.6	1.45 (0.47–4.47)	0.52		
PLR				
<150.6	1			
≥150.6	0.81 (0.27–2.40)	0.70		
LMR				
<3.36	1			
≥3.36	1.22 (0.41–3.65)	0.72		
PNI				
<52.35	1			
≥52.35	1.22 (0.41–3.65)	0.72		
SII				
<373.8	1			
≥373.8	0.78 (0.17–3.55)	0.75		
SIRI				
<0.94	1		1	
≥0.94	2.82 (0.90–8.85)	0.075	3.13 (1.01–9.71)	0.048
PIV				
<232.8	1			
≥232.8	0.48 (0.16–1.46)	0.20		

Abbreviations: CI, confidence interval; LMR, lymphocyte‐to‐monocyte ratio; NLR, neutrophil‐to‐lymphocyte ratio; PFS, progression‐free survival; PIV, pan‐immune inflammation value; PLR, platelet‐to‐lymphocyte ratio; PNI, prognostic nutrition index; SII, systemic immune‐inflammation index; SIRI, systemic inflammation response index.

### Treatment complications

Of the 51 patients available with acute toxicity following neoadjuvant treatment, the most common hematological toxicities were leukocytopenia and lymphopenia (Table 5). Two patients experienced grade III leukocytopenia and one patient experienced grade IV leukocytopenia. A total of 17 patients experienced grade III lymphopenia and one patient experienced grade IV lymphopenia. The most common nonhematological toxicity was esophagitis (grade 1: 31.4% and grade 2: 19.6%). Additionally, six patients experienced grade I pneumonia.

**TABLE 5:** Treatment toxicities of neoadjuvant CRT and pembrolizumab.

Toxicities	NCI‐CTC grade (*n* = 51)											
	0		1		2		3		4		5	
	No	%	No	%	No	%	No	%	No	%	No	%
Hematological												
Leukocytopenia	6	11.8	11	21.6	31	60.8	2	3.9	0	0	0	0
Neutropenia	17	33.3	21	41.2	9	17.6	2	3.9	1	0	0	0
Anemia	18	35.3	26	51	7	13.7	0	0	0	0	0	0
Thrombocytopenia	33	64.7	2	3.9	12	23.5	4	7.8	0	0	0	0
Glutamic pyruvic transaminase increased	46	90.2	2	3.9	2	3.9	1	2	0	0	0	0
Glutamic oxaloacetic transaminase increased	46	90.2	2	3.9	2	3.9	1	2	0	0	0	0
Nonhematological												
Esophagitis	25	49	16	31.4	10	19.6	0	0	0	0	0	0
Pneumonitis	45	88.2	6	11.8	0	0	0	0	0	0	0	0
Fatigue	40	78.4	8	15.7	3	5.9	0	0	0	0	0	0
Immune dermatitis	49	96.1	1	2	0	0	1	2	0	0	0	0

## DISCUSSION

During the past decade, the introduction of ICIs for the treatment of cancer has significantly changed the clinical treatment practice of solid tumors, including ESCC. The ATTRACTION‐3 trial^32^ demonstrated that nivolumab as second‐line therapy for ESCC significantly improved long‐term OS compared with chemotherapy (median, 10.9 vs. 8.5 months; HR, 0.79; *p* = 0.0264), with three‐year OS rates of 15.3% and 8.7%, respectively. Similarly, KEYNOTE‐181^33^ showed that pembrolizumab prolonged OS versus chemotherapy as second‐line therapy for advanced esophageal cancer in patients with PD‐L1 CPS ≥10 (median, 9.3 vs. 6.7 months; HR, 0.69, *p* = 0.0074). More recently, KEYNOTE‐590^34^ found that pembrolizumab plus chemotherapy improved OS when compared with placebo plus chemotherapy as first‐line therapy for esophageal cancer with PD‐L1 CPS of 10 or more (median 13·9 vs. 8·8 months; HR 0·57, *p* < 0.0001). Recently, immunotherapy has gained increasing attention in the era of neoadjuvant treatment of ESCC, and several clinical trials have also reported a pCR with 25%–55.6%. However, the role of ICIs in the treatment for resectable EC remains unknown in the era of neo‐CRT.

Immunotherapy has gained increasing attention in the era of neoadjuvant treatment of ESCC, and several clinical trials have also reported pCR with 25%–55.6%.^35^, ^36^, ^37^, ^38^ As a result, our team performed PALACE trials to investigate the efficacy and toxicities of neo‐CRT combined with pembrolizumab in patients with locally advanced ESCC. In the present study of 51 patients, 35 patients achieved pCR (68.6%), which is encouraging. However, the clinical benefits obtained from neo‐CRT and ICI therapy is not universal for all EC patients. Therefore, there is an urgent need to identify the optimal candidate patients who could most benefit from neo‐CRT combined ICI therapy.

Prior to the present study, multiple studies have demonstrated that various clinical IBs, such as NLR, LMR, and PLR, are significantly associated with clinical outcomes in numerous types of cancer, as cancer‐related inflammation plays an important role in cancer development and disease progression.^38^ Recent research has focused on the potential predictor for cancer patients treated with ICIs, and several inflammatory indexes, such as the NLR,^39^, ^40^ nutritional index,^41^ LMR,^42^ have also been reported as potential predictors of the effectiveness of anti‐PD‐1 antibody therapy. However, the prognostic of inflammatory and nutritional indexes in ESCC treated with neo‐CRT and ICIs remains unknown. In the present study, we demonstrate that a higher level of baseline peripheral ALC indicates a better survival outcome and archiving pCR when compared to low level of ALC among ESCC patients treated with neo‐CRT and pembrolizumab. One possible explanation for this finding is that lymphocytes play an essential role in mediating cellular immunity against tumor cells. A high level of lymphocytes could enhance the lymphocyte‐medicated antitumor immune response when concurrently used with pembrolizumab. Another possible explanation is that pretreatment ALC might be associated with disease status. Van Rossum et al. reported that patients with a lower baseline ALC were found to have the highest probability of developing grade 4 lymphopenia during CRT for esophageal cancer.^43^ Ray‐Coquard et al. reported that lymphopenia was significantly more frequent (*p* < 0.05) in metastatic breast cancer patients with performance status of >1.^44^ Fogar et al. found that lower circulating lymphocytes were found in patients with advanced pancreatic cancer who presented at advanced stages (stage IIB–IV, *p* < 0.05) compared with stages 0 to IIA.^45^ Based on our findings, for patients with baseline low level of ALC, radiation oncologists should pay more attention to the radiation field in order to protect the lymphocytes and the host immune system. In the present study, all patients were treated with involved field irradiation (IFI), CTV consisting of GTV with 3‐cm craniocaudal margin and only the malignant node is irradiated in order to decrease irradiation volume and achieve a pCR of 68.4%. Consistent with previous findings,^46^, ^47^ we also found that cT stage and baseline PLT count are two independent predictors for achieving pCR among ESCC patients treated with neo‐CRT and pembrolizumab.

The major strength of the present study is that it is an analysis based on a prospective cohort. Therefore, all patients included were treated with the predefined neo‐CRT with pembrolizumab according to the trial protocol. However, there are several limitations. First, despite the prospective nature of the included patients, this was a retrospective analysis of the prospective trial and baseline IBs were not the primary endpoints for analysis. Second, the sample size is small, and patients might be highly selected and from a single center; therefore, these factors may have restricted the generalizability of our results. Third, the median follow‐up time for the present study is 20 months. Therefore, the 2‐year survival outcomes of the present study might be inadequate, and the long‐term outcomes should be reported in order to confirm our findings. Finally, the cutoff value of ALC might vary among different studies and further studies are still needed to confirm our findings and identify the optimal value of baseline ALC value.

In conclusion, the present study demonstrates that pretreatment ALC is an independent predictor for achieving pCR and favorable outcomes among ESCC treated with neo‐CRT and pembrolizumab. We also determined that clinical T stage and baseline PLT are two independent predictors for achieving pCR in ESCC patients treated with neo‐CRT and pembrolizumab. Further multicenter and larger clinical trials should be performed to confirm our findings.

## AUTHOR CONTRIBUTIONS

Conceptualization: Jiayi Chen, Shengguang Zhao and Hecheng Li; Project administration: Jiayi Chen and Shengguang Zhao; Methodology: Wei‐Xiang Qi, Shengguang Zhao, Jiayi Chen; Data Curation: Xiaoyan Wang, Huan Li, and Shuyan Li; Formal analysis: Wei‐Xiang Qi. Manuscript preparation: Wei‐Xiang Qi, Chengqiang Li, Huan Li, Jiayi Chen, Shengguang Zhao prepared Figures 1–5: Feifei Xu and Xiaoyan Wang. All authors reviewed the manuscript; Final approval of manuscript: all authors.

## FUNDING INFORMATION

This study was supported by Clinical Research Plan of SHDC (SHDC2020CR2052B, SHDC2020CR4070); Special construction of integrated Chinese and Western medicine in general hospital (ZHYY‐ZXYJHZ X‐2‐201913).

## CONFLICT OF INTEREST STATEMENT

The author declares that they have no conflict of interest.

## INFORMED CONSENT

Informed consent was obtained from all individual participants included in the study.

## Data Availability

Raw data supporting the findings of this study are available from the corresponding author (S.Z.) on request.

## References

[1] Sung H , Ferlay J , Siegel RL , Laversanne M , Soerjomataram I , Jemal A , et al. Global cancer statistics 2020: GLOBOCAN estimates of incidence and mortality worldwide for 36 cancers in 185 countries. CA Cancer J Clin. 2021;71:209–49. 33538338 10.3322/caac.21660

[2] Shapiro J , van Lanschot JJB , Hulshof M , van Hagen P , van Berge Henegouwen MI , Wijnhoven BPL , et al. Neoadjuvant chemoradiotherapy plus surgery versus surgery alone for oesophageal or junctional cancer (CROSS): long‐term results of a randomised controlled trial, the lancet. Oncology. 2015;16:1090–8. 26254683 10.1016/S1470-2045(15)00040-6

[3] van Hagen P , Hulshof MC , van Lanschot JJ , Steyerberg EW , van Berge Henegouwen MI , Wijnhoven BP , et al. Preoperative chemoradiotherapy for esophageal or junctional cancer. New Engl J Med. 2012;366:2074–84. 22646630 10.1056/NEJMoa1112088

[4] Yang H , Liu H , Chen Y , Zhu C , Fang W , Yu Z , et al. Long‐term efficacy of neoadjuvant chemoradiotherapy plus surgery for the treatment of locally advanced esophageal squamous cell carcinoma: the NEOCRTEC5010 randomized clinical trial. JAMA Surgery. 2021;156:721–29. 34160577 10.1001/jamasurg.2021.2373 PMC8223138

[5] Yang H , Liu H , Chen Y , Zhu C , Fang W , Yu Z , et al. Neoadjuvant Chemoradiotherapy followed by surgery versus surgery alone for locally advanced squamous cell carcinoma of the esophagus (NEOCRTEC5010): a phase III multicenter, randomized, open‐label clinical trial. Clin Oncol. 2018;36:2796–803. 10.1200/JCO.2018.79.1483 PMC6145832 30089078

[6] Eyck BM , van Lanschot JJB , Hulshof M , van der Wilk BJ , Shapiro J , van Hagen P , et al. Ten‐year outcome of Neoadjuvant Chemoradiotherapy plus surgery for esophageal cancer: the randomized controlled CROSS trial. Clin Oncol. 2021;39:1995–2004. 10.1200/JCO.20.03614 33891478

[7] Buque A , Bloy N , Aranda F , Castoldi F , Eggermont A , Cremer I , et al. Trial watch: immunomodulatory monoclonal antibodies for oncological indications. Onco Targets Ther. 2015;4:e1008814. 10.1080/2162402X.2015.1008814 PMC4485728 26137403

[8] Gou Q , Dong C , Xu H , Khan B , Jin J , Liu Q , et al. PD‐L1 degradation pathway and immunotherapy for cancer. Cell Death Dis. 2020;11:955. 33159034 10.1038/s41419-020-03140-2 PMC7648632

[9] Li C , Zhao S , Zheng Y , Han Y , Chen X , Cheng Z , et al. Preoperative pembrolizumab combined with chemoradiotherapy for oesophageal squamous cell carcinoma (PALACE‐1). Eur J Cancer. 2021;144:232–41. 33373868 10.1016/j.ejca.2020.11.039

[10] Feng JF , Huang Y , Chen QX . Preoperative platelet lymphocyte ratio (PLR) is superior to neutrophil lymphocyte ratio (NLR) as a predictive factor in patients with esophageal squamous cell carcinoma. World J Surg Oncol. 2014;12:58. 24641770 10.1186/1477-7819-12-58 PMC3973187

[11] Chen MF , Chen PT , Kuan FC , Chen WC . The predictive value of pretreatment neutrophil‐to‐lymphocyte ratio in esophageal squamous cell carcinoma. Ann Surg Oncol. 2019;26:190–9. 30362062 10.1245/s10434-018-6944-1

[12] Fu X , Li T , Dai Y , Li J . Preoperative systemic inflammation score (SIS) is superior to neutrophil to lymphocyte ratio (NLR) as a predicting indicator in patients with esophageal squamous cell carcinoma. BMC Cancer. 2019;19:721. 31331297 10.1186/s12885-019-5940-6 PMC6647281

[13] Guo Q , Shao Z , Xu D , Fan L , Xiong H , Ding X , et al. Prognostic value of neutrophil‐to‐lymphocyte ratio in peripheral blood and pathological tissue in patients with esophageal squamous cell carcinoma. Medicine. 2020;99:e21306. 32702926 10.1097/MD.0000000000021306 PMC7373615

[14] Hoshino S , Takeuchi M , Kawakubo H , Matsuda S , Mayanagi S , Irino T , et al. Usefulness of neutrophil to lymphocyte ratio at recurrence for predicting long‐term outcomes in patients with recurrent esophageal squamous cell carcinoma. Ann Surg Oncol. 2021;28:3001–8. 33689078 10.1245/s10434-021-09637-0

[15] Sun Y , Zhang L . The clinical use of pretreatment NLR, PLR, and LMR in patients with esophageal squamous cell carcinoma: evidence from a meta‐analysis. Cancer Manag Res. 2018;10:6167–79. 30538564 10.2147/CMAR.S171035 PMC6257133

[16] Zhi X , Jiang K , Shen Y , Su X , Wang K , Ma Y , et al. Peripheral blood cell count ratios are predictive biomarkers of clinical response and prognosis for non‐surgical esophageal squamous cell carcinoma patients treated with radiotherapy. J Clin Lab Anal. 2020;34:e23468. 32681567 10.1002/jcla.23468 PMC7595892

[17] Wang C , Tong J , Tang M , Lu Y , Liang G , Zhang Z , et al. Pretreatment neutrophil‐to‐lymphocyte ratio and platelet‐to‐lymphocyte ratio as prognostic factors and reference markers of treatment options for locally advanced squamous cell carcinoma located in the middle and upper esophagus. Cancer Manag Res. 2021;13:1075–85. 33574705 10.2147/CMAR.S294344 PMC7872927

[18] Cai G , Yu J , Meng X . Predicting prognosis and adverse events by hematologic markers in patients with locally advanced esophageal squamous cell carcinoma treated with Neoadjuvant Chemoradiotherapy. Cancer Manag Res. 2020;12:8497–507. 33061564 10.2147/CMAR.S257058 PMC7519412

[19] Shang QX , Yang YS , Hu WP , Yuan Y , He Y , Zhao JY , et al. Clinical and prognostic significance of preoperative lymphocyte‐monocyte ratio, neutrophil‐lymphocyte ratio and neutrophil‐monocyte ratio on esophageal squamous cell carcinoma patients. Transl Cancer Res. 2020;9:3903–14. 35117757 10.21037/tcr-19-2777 PMC8797393

[20] Wu Y , Chen J , Zhao L , Li Q , Zhu J , Yang H , et al. Prediction of pathologic response to Neoadjuvant Chemoradiotherapy in patients with esophageal squamous cell carcinoma incorporating hematological biomarkers. Cancer Res Treatment. 2021;53:172–83. 10.4143/crt.2020.594 PMC7812014 32898941

[21] Wu X , Han R , Zhong Y , Weng N , Zhang A . Post treatment NLR is a predictor of response to immune checkpoint inhibitor therapy in patients with esophageal squamous cell carcinoma. Cancer Cell Int. 2021;21:356. 34233686 10.1186/s12935-021-02072-x PMC8262036

[22] Valero C , Lee M , Hoen D , Weiss K , Kelly DW , Adusumilli PS , et al. Pretreatment neutrophil‐to‐lymphocyte ratio and mutational burden as biomarkers of tumor response to immune checkpoint inhibitors. Nat Commun. 2021;12:729. 33526794 10.1038/s41467-021-20935-9 PMC7851155

[23] Xiao L , Li L , Chen G , Zhang Y , Gao Q . The lymphocyte‐to‐monocyte ratio could predict the efficacy of PD‐1 inhibitors in patients with advanced cancer, Transl. Cancer Res. 2020;9:4111–20. 10.21037/tcr-20-1451 PMC8799307 35117780

[24] Qi WX , Xiang Y , Zhao S , Chen J . Assessment of systematic inflammatory and nutritional indexes in extensive‐stage small‐cell lung cancer treated with first‐line chemotherapy and atezolizumab. Cancer Immunol Immunother. 2021;70:3199–206. 33796915 10.1007/s00262-021-02926-3 PMC10991671

[25] Shoji F , Takeoka H , Kozuma Y , Toyokawa G , Yamazaki K , Ichiki M , et al. Pretreatment prognostic nutritional index as a novel biomarker in non‐small cell lung cancer patients treated with immune checkpoint inhibitors. Lung Cancer. 2019;136:45–51. 31437663 10.1016/j.lungcan.2019.08.006

[26] De Giorgi U , Procopio G , Giannarelli D , Sabbatini R , Bearz A , Buti S , et al. Association of Systemic Inflammation Index and Body Mass Index with survival in patients with renal cell cancer treated with Nivolumab. Clin Cancer Res. 2019;25:3839–46. 30967420 10.1158/1078-0432.CCR-18-3661

[27] Bauckneht M , Genova C , Rossi G , Rijavec E , Dal Bello MG , Ferrarazzo G , et al. The role of the immune metabolic prognostic index in patients with non‐small cell lung cancer (NSCLC) in radiological progression during treatment with Nivolumab. Cancers. 2021;13:3117. 34206545 10.3390/cancers13133117 PMC8268031

[28] Zhao M , Duan X , Han X , Wang J , Han G , Mi L , et al. Sarcopenia and systemic inflammation response index predict response to systemic therapy for hepatocellular carcinoma and are associated with immune cells. Front Oncol. 2022;12:854096. 35463384 10.3389/fonc.2022.854096 PMC9024177

[29] Corti F , Lonardi S , Intini R , Salati M , Fenocchio E , Belli C , et al. The Pan‐immune‐inflammation value in microsatellite instability‐high metastatic colorectal cancer patients treated with immune checkpoint inhibitors. Eur J Cancer. 2021;150:155–67. 33901794 10.1016/j.ejca.2021.03.043

[30] Zeng R , Liu F , Fang C , Yang J , Luo L , Yue P , et al. PIV and PILE score at baseline predict clinical outcome of anti‐PD‐1/PD‐L1 inhibitor combined with chemotherapy in extensive‐stage small cell lung cancer patients. Front Immunol. 2021;12:724443. 34777341 10.3389/fimmu.2021.724443 PMC8586214

[31] Cortellini A , Bersanelli M , Santini D , Buti S , Tiseo M , Cannita K , et al. Another side of the association between body mass index (BMI) and clinical outcomes of cancer patients receiving programmed cell death protein‐1 (PD‐1)/ programmed cell death‐ligand 1 (PD‐L1) checkpoint inhibitors: a multicentre analysis of immune‐related adverse events. Eur J Cancer. 2020;128:17–26. 32109847 10.1016/j.ejca.2019.12.031

[32] Okada M , Kato K , Cho BC , Takahashi M , Lin CY , Chin K , et al. Three‐year follow‐up and response‐survival relationship of nivolumab in previously treated patients with advanced esophageal squamous cell carcinoma (ATTRACTION‐3). Clin Cancer Res. 2022;28:3277–86. 35294546 10.1158/1078-0432.CCR-21-0985 PMC9662935

[33] Kojima T , Shah MA , Muro K , Francois E , Adenis A , Hsu CH , et al. K.‐. investigators, randomized phase III KEYNOTE‐181 study of Pembrolizumab versus chemotherapy in advanced esophageal cancer. Clin Oncol. 2020;38:4138–48. 10.1200/JCO.20.01888 33026938

[34] Sun JM , Shen L , Shah MA , Enzinger P , Adenis A , Doi T , et al. K.‐. investigators, Pembrolizumab plus chemotherapy versus chemotherapy alone for first‐line treatment of advanced oesophageal cancer (KEYNOTE‐590): a randomised, placebo‐controlled, phase 3 study. Lancet. 2021;398:759–71. 34454674 10.1016/S0140-6736(21)01234-4

[35] Liu J , Li J , Lin W , Shao D , Depypere L , Zhang Z , et al. Neoadjuvant camrelizumab plus chemotherapy for resectable, locally advanced esophageal squamous cell carcinoma (NIC‐ESCC2019): a multicenter, phase 2 study. Int J Cancer. 2022;151:128–37. 35188268 10.1002/ijc.33976

[36] Yan X , Duan H , Ni Y , Zhou Y , Wang X , Qi H , et al. Tislelizumab combined with chemotherapy as neoadjuvant therapy for surgically resectable esophageal cancer: a prospective, single‐arm, phase II study (TD‐NICE). Int J Surg. 2022;103:106680. 35595021 10.1016/j.ijsu.2022.106680

[37] Shen D , Chen Q , Wu J , Li J , Tao K , Jiang Y . The safety and efficacy of neoadjuvant PD‐1 inhibitor with chemotherapy for locally advanced esophageal squamous cell carcinoma. J Gastrointest Oncol. 2021;12:1–10. 33708420 10.21037/jgo-20-599 PMC7944149

[38] Mantovani A , Allavena P , Sica A , Balkwill F . Cancer‐related inflammation. Nature. 2008;454:436–44. 18650914 10.1038/nature07205

[39] Petrova MP , Eneva MI , Arabadjiev JI , Conev NV , Dimitrova EG , Koynov KD , et al. Neutrophil to lymphocyte ratio as a potential predictive marker for treatment with pembrolizumab as a second line treatment in patients with non‐small cell lung cancer. Biosci Trends. 2020;14:48–55. 32023563 10.5582/bst.2019.01279

[40] Matsubara T , Takamori S , Haratake N , Toyozawa R , Miura N , Shimokawa M , et al. The impact of immune‐inflammation‐nutritional parameters on the prognosis of non‐small cell lung cancer patients treated with atezolizumab. J Thorac Dis. 2020;12:1520–8. 32395289 10.21037/jtd.2020.02.27 PMC7212122

[41] Ohba T , Takamori S , Toyozawa R , Nosaki K , Umeyama Y , Haratake N , et al. Prognostic impact of the controlling nutritional status score in patients with non‐small cell lung cancer treated with pembrolizumab. J Thorac Dis. 2019;11:3757–68. 31656648 10.21037/jtd.2019.09.29 PMC6790442

[42] Failing JJ , Yan Y , Porrata LF , Markovic SN . Lymphocyte‐to‐monocyte ratio is associated with survival in pembrolizumab‐treated metastatic melanoma patients. Melanoma Res. 2017;27:596–600. 29016387 10.1097/CMR.0000000000000404

[43] van Rossum PSN , Deng W , Routman DM , Liu AY , Xu C , Shiraishi Y , et al. Prediction of severe Lymphopenia during chemoradiation therapy for esophageal cancer: development and validation of a pretreatment nomogram. Pract Radiat Oncol. 2020;10:e16–26. 31369887 10.1016/j.prro.2019.07.010 PMC7564893

[44] Ray‐Coquard I , Cropet C , Van Glabbeke M , Sebban C , Le Cesne A , Judson I , et al. Bone sarcoma, Lymphopenia as a prognostic factor for overall survival in advanced carcinomas, sarcomas, and lymphomas. Cancer Res. 2009;69:5383–91. 19549917 10.1158/0008-5472.CAN-08-3845 PMC2775079

[45] Fogar P , Sperti C , Basso D , Sanzari MC , Greco E , Davoli C , et al. Decreased total lymphocyte counts in pancreatic cancer: an index of adverse outcome. Pancreas. 2006;32:22–8. 16340740 10.1097/01.mpa.0000188305.90290.50

[46] Xie J , Pang Y , Li X , Wu X . The log odds of negative lymph nodes/T stage: a new prognostic and predictive tool for resected gastric cancer patients. J Cancer Res Clin Oncol. 2021;147:2259–69. 34003367 10.1007/s00432-021-03654-y PMC8236481

[47] Deng J , Chu X , Ren Z , Wang B . Relationship between T stage and survival in distantly metastatic esophageal cancer: a STROBE‐compliant study. Medicine. 2020;99:e20064. 32384472 10.1097/MD.0000000000020064 PMC7220676

